# Litholapaxy

**Published:** 1886-12

**Authors:** 


*Litholapaxy.* By P. J. FREYER, M.A., M.D., M.Ch., Bengal Med. Service. Pp. 115. London: J. & A. CHURCHILL.

We lay down this work fully believing that the author will realise his hope "that the record of a large number of cases of this operation, from the practice of a single operator, may prove interesting to the profession at large, and that the results obtained may have some effect in bringing into more general practice . . . an operation . . . which has a brilliant future before it." Dr. Freyer lost only five out of 128 cases—and one of these was from bronchitis and exposure. If anything more than this brilliant result could popularise an operation, the clear, simple, practical narrative before us would.

We cannot refrain from expressing our sympathy with Dr. Freyer in his criticism of Sir Henry Thompson's behaviour and language with regard to this operation. With dignity and equability of temper, the author condemns Sir H. Thompson out of his own mouth. We agree with Dr. Freyer, that Bigelow's operation was entirely original, that it was a grand revolution in surgical proceeding, and that, even in the uncouth name Litholapaxy, Bigelow was more than justified in the independent position which he took up. We would further express our agreement by saying that Sir Henry Thompson undoubtedly did condemn Bigelow's operation at the outset; that he subsequently sought to prove that he had been leading up to it, even doing it, for years; that he introduced a modification of Bigelow's instruments (after condemning one or two of them); and that now he writes of it with an implied sense of proprietorship, not as Bigelow's operation, or as litholapaxy, but as "lithotrity at one sitting." Probably it is no use protesting that the lithotrity is not the only or even the major part of the operation; still, we are pleased to observe that Sir Henry's fathering of the proceeding has not done away with Bigelow's name. And we are pleased to find that our practical and experienced *confrère* in India has dared to speak out what we know to have been in the minds of many English surgeons as to this attempt by our leading authority on calculus to minimise the reputation of an American brother.

